# Asymptomatic *Plasmodium* infections in 18 villages of southern Savannakhet Province, Lao PDR (Laos)

**DOI:** 10.1186/s12936-016-1336-0

**Published:** 2016-05-27

**Authors:** Koukeo Phommasone, Bipin Adhikari, Gisela Henriques, Tiengkham Pongvongsa, Panom Phongmany, Lorenz von Seidlein, Nicholas J. White, Nicholas P. J. Day, Arjen M. Dondorp, Paul N. Newton, Mallika Imwong, Mayfong Mayxay

**Affiliations:** Microbiology Laboratory, Lao-Oxford-Mahosot Hospital-Wellcome Trust Research Unit (LOMWRU), Vientiane, Lao PDR; Mahidol Oxford Tropical Medicine Research Unit (MORU), Faculty of Tropical Medicine, Mahidol University, Bangkok, Thailand; Savannakhet Provincial Health Department, Nakhon Sawan, Savannakhet Province Lao PDR; Centre for Tropical Medicine and Global Health, Nuffield Department of Clinical Medicine, University of Oxford, Oxford, United Kingdom; Faculty of Postgraduate Studies, University of Health Sciences, Vientiane, Lao PDR

**Keywords:** Asymptomatic, RDT, uPCR, Prevalence, Lao PDR

## Abstract

**Background:**

A large fraction of *Plasmodium* infections do not cause clinical signs and symptoms of disease and persist at densities in blood that are not detectable by microscopy or rapid diagnostic tests. These infections may be critical as a transmission reservoir in areas of low malaria endemicity. Understanding the epidemiology of these infections would be helpful for malaria elimination.

**Methods:**

A cross-sectional survey was conducted in Thapangthong and Nong Districts of Savannakhet Province, Lao PDR, to determine the prevalence of parasitaemia. A total of 888 blood samples were collected from afebrile volunteers aged ≥15 years in 18 villages during March and July 2015. *Plasmodium* infections were diagnosed by rapid diagnostic tests (RDT) and high volume, ultra-sensitive quantitative polymerase chain reaction (uPCR).

**Results:**

uPCR detected *Plasmodium* infections in 175 of 888 samples (20 %). The species distribution was *Plasmodiumfalciparum* 3.6 % (32/888), *Plasmodium vivax* 11.1 % (99/888), mixed infections with *P. falciparum* and *P. vivax* 1.6 % (14/888) and *Plasmodium* of undetermined species 3.4 % (30/888). RDT identified only 2 % (18/888) positive cases. Using uPCR as reference, the sensitivity and specificity of RDTs were 28 and 100 %, respectively, in detecting *P. falciparum* infections, and 3 and 99 % in detecting asymptomatic *P. vivax* infections. The K13 kelch propeller domain C580Y mutation, associated with reduced susceptibility to artemisinin derivatives, was found in 75 % (12/18) of *P. falciparum* isolates from Thapangthong and in 7 % (2/28) from Nong (p < 0.001). In a multivariate analysis, males were more likely to have *P. vivax* infections [adjusted odds ratio (aOR) 4.76 (95 % CI 2.84–8.00)] while older villagers were at lower risk for parasitaemia [aOR for increasing age 0.98 (95 % CI 0.96–0.99)].

**Conclusion:**

There is a high prevalence of asymptomatic *Plasmodium* infections in southern Savannakhet. Artemisinin-resistant *P. falciparum* strains form an increasing proportion of the parasite population in Thapangthong District and are already present in the more remote Nong District. This worrying trend has wider implications for Laos and could reverse the gains achieved by the successful control of malaria in Laos and the Greater Mekong Sub-region (GMS). Rapid elimination of *P. falciparum* has to be a top priority in Laos as well as in the wider GMS.

## Background

Substantial progress has been made in the control of malaria in the Lao PDR (Laos), in particular in the north of the country. The five southernmost provinces, Savannakhet, Salavan, Sekong, Champasack, and Attapeu accounted for 90 % of all malaria patients reported in the country in 2008 [1]. A nationwide malaria survey conducted between 2006 and 2008, including 495 health centres in Savannakhet Province, reported an overall incidence of 11.5 *Plasmodium falciparum* cases per 1000 people. The survey ranked Savannakhet as the province with the third highest *P. falciparum* cases recorded in 2008 [2].

In Laos, many remote health centres rely on rapid diagnostic tests (RDT) for malaria diagnosis. Few regional and district-level health centres have access to microscopy [3]. The nationwide prevalence survey carried out during 2006–2008 was based on passive case reporting by provincial and district hospitals, provincial malaria stations, health centres, and village health workers (VHWs). Case detection was based on either RDTs or microscopy [1, 2]. The majority of malaria infections remained undetected since only symptomatic cases were captured [4–7]. People with asymptomatic *Plasmodium* infections can carry very low parasite densities, for extended periods, which are undetectable by microscopy or RDTs [5, 6]. Mosquitoes feeding on blood samples from individuals with sub-microscopic *Plasmodium* infections can become infected [8, 9]. Thus, sub-microscopic carriers contribute to malaria transmission [10, 11].

The elimination of malaria in the greater Mekong Sub-region (GMS) has become particularly urgent with the emergence and spread of artemisinin resistance, the failure of artemisinin combination therapy (ACT) partner drugs, and the threat of untreatable malaria [12]. Current recommendations to prevent further spread of drug-resistant malaria from Southeast Asia advocate regional malaria elimination [13, 14]. As a part of National Strategic Plan for Malaria Control and Elimination 2011–2015, Laos has adopted the goal of eliminating malaria by 2030 [1, 15].

To gain a better understanding of which villages need to be targeted for malaria elimination, a survey was conducted in 18 villages of southern Savannakhet Province, which has a historically high malaria prevalence based on village malaria worker records.

## Methods

### Study site and design

**Fig. 1:**
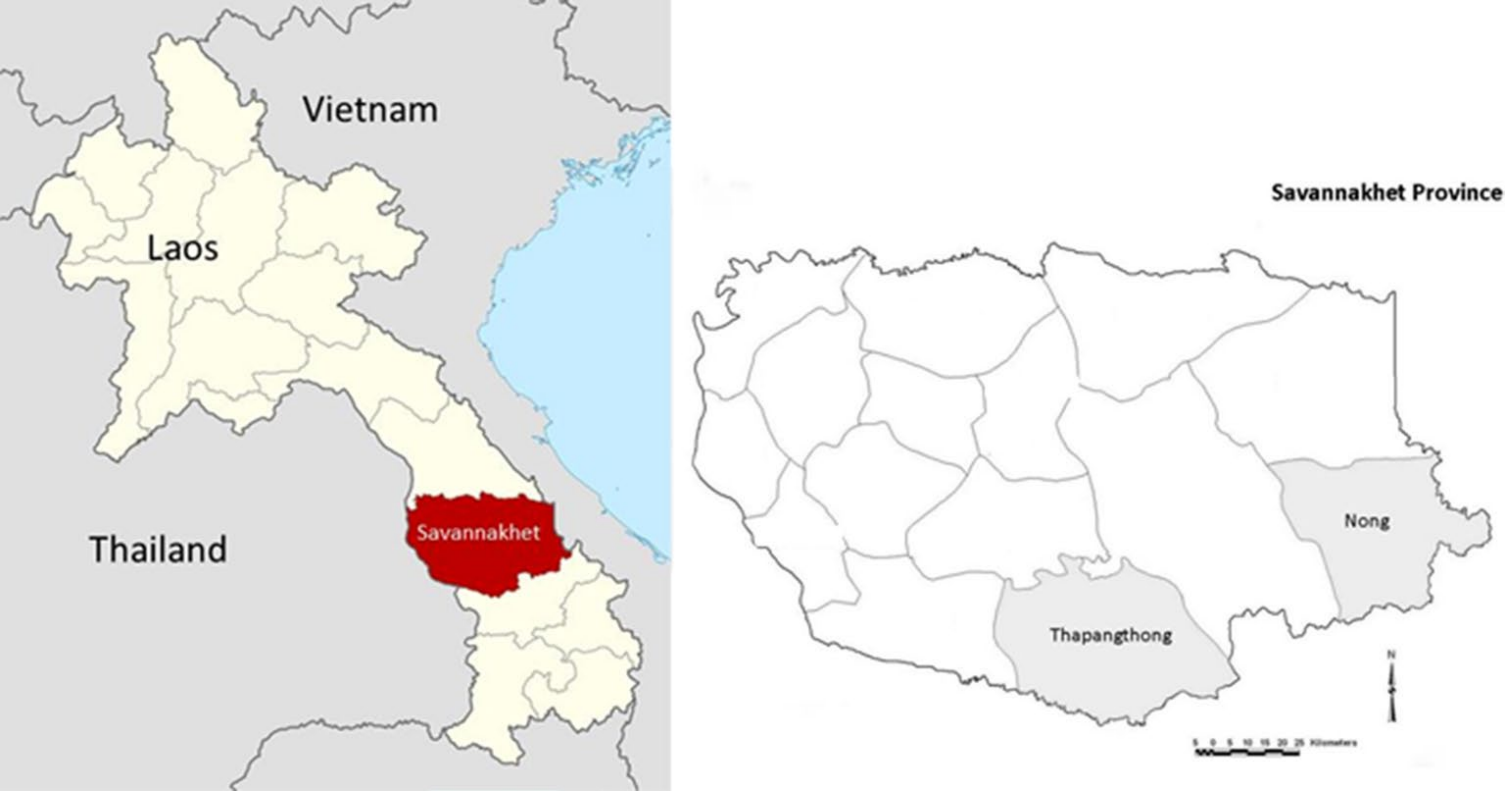
Study sites within Savannakhet Province

The study was conducted in southern Savannakhet Province, Laos. The province is ~600 km south from Vientiane, the capital city of Laos. It has a total area of 21,774 km^2^ and includes 15 districts [16]. Savannakhet is the most populous province of Laos with a total population of ~843,245 people, representing about 14 % of the population of the country. The province has one provincial hospital, 15 district hospitals and 115 health centres. This health system covers approximately 89 % of the province’s geographical area [17]. Cross-sectional surveys were conducted in Thapangthong and Nong Districts (see Fig. 1). The districts and villages were chosen based on the previous high malaria incidence in provincial epidemiological records. Villagers were informed by local health centre staff of the reasons for the survey and requested to arrive at a suitable location within each village. A mobile study team with blood collection equipment, tools for anthropometry and essential medicines conducted the study in each village.

### Study participants and procedures

A description of the study was announced at village meetings. Additional explanations about the study were provided to each participant during the consent process before blood sample collection. Volunteers of age ≥15 years were enrolled into the study. Written consent was obtained from each volunteer before participation. Travel costs were reimbursed and vitamin B complex and/or haematinics were given to the study participants based on the judgment of study clinicians. Information on demographics (age, sex, weight, height), tympanic temperature, history of fever and history of illness during the previous 48 h, a history of malaria, anti-malarial drug treatment, recent travel, and bed net use was collected using the Open Data Kit (ODK^®^) application on a smartphone.

### Sample collection

Villagers who met the inclusion criteria were asked to give 3 mL venous blood samples which were collected into EDTA tubes and stored in ice pack cooling boxes until their transportation to a centralized field laboratory in the two districts (within 6 h of blood collection). Upon return to the centralized laboratory, the whole blood was separated and the red blood cell pellets were frozen promptly and stored at −20 °C for up to 7 days. Each sample was labelled with a barcode to ensure blinding and negative controls were added to the sample pool. The samples were transported on dry ice to the molecular parasitology laboratory in Bangkok, Thailand for analysis.

All participants were tested on site for malaria using the SD Bioline Ag Pf/Pan (Standard Diagnostics Inc) RDT. Those with positive RDTs were treated with artemether/lumefantrine, as per Laos national treatment guidelines. The RDT test was performed and interpreted by an experienced laboratory technician according to manufacturer’s recommendation.

### DNA extraction and PCR amplification

DNA was extracted from thawed packed red blood cells using an automated DNA extraction machine (QIAsymphony and DPS DNA midi kit; Qiagen, Germany). DNA was dried, concentrated and then used as a template for PCR detection and quantification of *Plasmodium*. Quantitative ultrasensitive PCR (uPCR) analysis was performed as described elsewhere [18]. Briefly, DNA of *Plasmodium* was detected and quantified using 18S rRNA-targeting primers. The limit of detection was ~22 parasites/mL. For *Plasmodium*-positive samples, an attempt was made to identify the species using *P. falciparum*- and *Plasmodium vivax*-specific PCR protocols as described previously [18].

To detect polymorphisms associated with reduced susceptibility to artemisinin derivatives, the open-reading frame of the PF3D7_1343700 kelch propeller domain was amplified using a nested PCR protocol [12, 19]. Purified PCR products were sequenced at Macrogen, Republic of Korea, and analysed using BioEdit version 7.1.3.0., using the 3D7 *kelch13* sequence as reference (Accession: XM_001350122.1). The definition of single nucleotide polymorphisms (SNPs) was based on analytical approaches described previously [12, 20].

### Statistical analysis

Asymptomatic malaria was defined as a *Plasmodium* infection detected in study participants who were afebrile (tympanic temperature of <37.5 °C) at the time of the survey and had no history of fever in the preceding 48 h. Sub-microscopic infections were here defined as a *Plasmodium* infection with densities too low to be detectable by microscopy and malaria RDT but detectable by uPCR.

Descriptive statistics were used to analyse the baseline characteristics of the study population. The Chi squared test and Kruskal–Wallis test were used to test associations between variables and *Plasmodium* infection in univariate analyses. Multiple logistic regression models were constructed and used to explore associations between *P. falciparum* mono-infections and independent co-variables as well as for *P. vivax* mono-infections and independent co-variables. Variables which were significant at the P < 0.05 level in the univariate analysis were evaluated in a multivariable regression model. The models were run for any *Plasmodium* infections and then for *P. falciparum* and *P. vivax* as dependent variables. P values less than 0.05 were considered statistically significant. Statistical analysis was performed using STATA 14.0 (StataCorp, College Station, TX, USA).

### Ethics statement

Ethical approval for the study was received from the Lao National Ethics Committee for Health Research (Ref No 013-2015/NECHR), Government of the Laos and the Oxford Tropical Research Ethics Committee (1015-13). Individual informed consent was obtained from each participant.

## Results

A total of 888 volunteers, 433 from eight villages in Thapangthong District and 455 from ten villages in Nong District participated in the study. The surveys were conducted between 21 and 24 March, 2015 in Thapangthong District and between 22 and 26 July, 2015 in Nong District.

### Demographic characteristics of participants

The overall median age was 33 years (IQR 22–47 years). More than half (57 %) of the participants were in the age range between 15 and 35 years and 479/888 (54 %) were male. Only two among 888 participants (0.2 %) had a fever (≥37.5 °C) at the time of the survey (Table 1). One febrile participant (39.3 °C) was blood smear- and RDT-negative but was later found to be infected by *P. vivax* using uPCR. The other participant (37.6 °C) was not parasitaemic. The mean weight and height of participants were 48 kg (SD = 7.6) and 153 cm (SD = 7.3), respectively.

**Table 1:** Baseline characteristics of the study population (n = 888)

Characteristics	Results					P value**
	Negative	Positive				
		*P. falciparum*	*P. vivax*	Mixed (*Pf* + *Pv*)^a^	*Plasmodium* spp.	
Sex						
Male (n = 479)	354 (74 %)	16 (3 %)	76 (16 %)	10 (2 %)	23 (5 %)	<0.001
Female (n = 409)	359 (88 %)	16 (4 %)	23 (6 %)	4 (1 %)	7 (2 %)	
Age group						
15–35 years (n = 505)	383 (76 %)	22 (4 %)	73 (15 %)	8 (2 %)	19 (4 %)	0.009
36–55 years (n = 234)	196 (84 %)	7 (3 %)	20 (9 %)	5 (2 %)	6 (3 %)	
56–80 years (n = 149)	134 (90 %)	3 (2 %)	6 (4 %)	1 (0.7 %)	5 (3 %)	
Mean age in years (n = 888; SD)	37.0 (16.2)	32.0 (13.9)	30.9 (13.6)	32.9 (13.9)	34.7 (15.7)	0.005
History of Fever in the last 48 h						
Yes (n = 277)	223 (80 %)	17 (6 %)	26 (9 %)	4 (1 %)	7 (3 %)	0.056
No (n = 611)	490 (80 %)	15 (3 %)	73 (12 %)	10 (2 %)	23 (4 %)	
Feeling ill in the last 48 h						
Yes (n = 451)	370 (82 %)	20 (4 %)	43 (10 %)	5 (1 %)	13 (3 %)	0.186
No (n = 437)	343 (79 %)	12 (3 %)	56 (13 %)	9 (2 %)	17 (4 %)	
History of malaria						
Yes (n = 493)	367 (74 %)	21 (4 %)	76 (15 %)	10 (2 %)	19 (4 %)	<0.001
No (n = 385)	336 (87 %)	11 (3 %)	23 (6 %)	4 (1 %)	11(3 %)	
Episodes of malaria						
0 Episodes (n = 395)	346 (88 %)	11 (3 %)	23 (6 %)	4 (1 %)	11 (3 %)	<0.001
1–3 episodes (n = 409)	320 (78 %)	20 (5 %)	47 (12 %)	7 (2 %)	15 (4 %)	
>3 episodes (n = 84)	47 (56 %)	1 (1 %)	29 (35 %)	3 (4 %)	4 (5 %)	
Stay overnight in the forest in the last 3 months^a^						
<2 weeks ago (n = 183)	136 (74 %)	7 (4 %)	30 (16 %)	3 (2 %)	7 (4 %)	<0.001
2–4 weeks ago (n = 27)	16 (59 %)	1 (4 %)	8 (30 %)	1 (4 %)	1 (4 %)	
>4 weeks ago (n = 66)	43 (65 %)	1 (2 %)	15 (23 %)	4 (6 %)	3 (5 %)	
No forest visits (n = 612)	518 (85 %)	23 (4 %)	46 (8 %)	6 (1 %)	19 (3 %)	
Travelled outside the village in the last 3 months^a^						
Yes (n = 232)	188 (81 %)	10 (4 %)	23 (10 %)	4 (2 %)	7 (3 %)	
No (n = 656)	525 (80 %)	22 (3 %)	76 (12 %)	10 (2 %)	23 (4 %)	0.902
Bed net use						
Every day (n = 682)	559 (82 %)	26 (4 %)	67 (10 %)	8 (1 %)	22 (3 %)	0.132
Sometimes (n = 143)	108 (76 %)	3 (2 %)	24 (17 %)	4 (3 %)	4 (3 %)	
Never (n = 63)	46 (73 %)	3 (5 %)	8 (13 %)	2 (3 %)	4 (6 %)	

^a^Mixed infection with *P.falciparum* and *P. vivax*

** Chi squared test

### Prevalence of *Plasmodium* infections

Overall, *Plasmodium* parasites were detected in 175/888 individuals (20 %) by uPCR (Table 2): 99/888 (11 %) had *P. vivax* mono-infections, 32/888 (4 %) had *P. falciparum* mono-infections and 14/888 (2 %) had mixed infections (*P. vivax* and *P. falciparum*). The *Plasmodium* species could not be determined in specimens from 30/888 (3.4 %) patients. The prevalence of *P. vivax* mono-infections was significantly lower in Nong [5 % (22/455)] than in Thapangthong [18 % (77/433); p < 0.0001]. In contrast, the prevalence of *P. falciparum* mono-infections was higher in Nong [5 % (24/455)] compared to Thapangthong [2 % (8/433); p = 0.1; Table 2]. There was also high variability in *Plasmodium* prevalence within districts. In the eight villages in Thapangthong District the *Plasmodium* infection prevalence ranged between 12 and 39 % and in the ten villages in Nong District between 4 and 56 %.

**Table 2:** Parasite prevalence detected by uPCR by village and district (n = 888)

Villages	Negative	Positive				PfK-13 resistance Marker C580Y
		*PF*	*PF* + *PV*	*PV*	*P species*	
Thapangthong District						
Maiphosy (n = 50)	38 (76 %)	0	1 (2 %)	11 (22 %)	0	1/1 (100 %)
Bouttaphan (n = 59)	42 (71 %)	1 (2 %)	2 (3 %)	14 (24 %)	0	3/3 (100 %)
Maixe (n = 58)	47 (81 %)	2 (3 %)	0	7 (12 %)	2 (3 %)	1/2 (50 %)
Phouphanang (n = 56)	37 (66 %)	2 (4 %)	1 (2 %)	15 (27 %)	1 (2 %)	3/3 (100 %)
Phoumaly (n = 52)	42 (81 %)	1 (2 %)	1 (2 %)	6 (12 %)	2 (4 %)	2/2 (100 %)
Nalao (n = 52)	40 (77 %)	1 (2 %)	2 (4 %)	7 (13 %)	2 (4 %)	0/3
Khampia (n = 49)	30 (61 %)	1 (2 %)	1 (2 %)	14 (29 %)	3 (6 %)	2/2 (100 %)
Kengkhai (n = 57)	50 (88 %)	0	0	3 (5 %)	4 (7 %)	0/0
Thapangthong Total (n = 433)	326 (75 %)	8 (2 %)	8 (2 %)	77 (18 %)	14 (3 %)	12/16 (75 %)
Nong District						
Oi Tan Tip (n = 50)	22 (44 %)	4 (8 %)	5 (10 %)	15 (30 %)	4 (8 %)	0/8
Denvilay (n = 50)	46 (92 %)	1 (2 %)	0	2 (4 %)	1 (2 %)	0/1
Asing na (n = 19)	15 (79 %)	1 (5 %)	0	2 (11 %)	1 (5 %)	0/1
Asing saly (n = 29)	27 (93 %)	0	0	0	2 (7 %)	0/0
Phounmakmy (n = 57)	46 (81 %)	9 (16 %)	0	0	2 (4 %)	0/8
Kaysone (n = 50)	46 (92 %)	2 (4 %)	0	1 (2 %)	1 (2 %)	0/1
Thathe (n = 49)	45 (92 %)	1 (2 %)	1 (2 %)	1 (2 %)	1 (2 %)	1/2 (50 %)
Xuangtai (n = 50)	44 (88 %)	3 (6 %)	0	0	3 (6 %)	0/1
Paloy (n = 47)	45 (96 %)	1 (2 %)	0	1 (2 %)	0	1/1 (100 %)
Salang (n = 54)	51 (94 %)	2 (4 %)	0	0	1 (2 %)	0/2
Nong Total (n = 455)	387 (85 %)	24 (5 %)	6 (1 %)	22 (5 %)	16 (4 %)	2/28 (7 %)
Overall Total (n = 888)	713 (80 %)	32 (4 %)	14 (2 %)	99 (11 %)	30 (3 %)	14/44 (32 %)

*Pf* *Plasmodium falciparum, Pv* *Plasmodium vivax*

### Resistance markers

The mutation C580Y in the PF3D7_1343700 kelch propeller domain, which is associated with reduced susceptibility to artemisinin derivatives, was found in 32 % (of 14/44) *P. falciparum* strains (Table 2). No other kelch polymorphisms were detected. The C580Y mutation was found in 75 % (12/18) of *P. falciparum* strains detected in Thapangthong and in 7 % (2/28) strains detected in Nong (p < 0.001).

### *Plasmodium* prevalence by RDT

RDTs detected eight participants (8/888; 0.9 %) with *P. falciparum* mono-infections, six participants (6/888; 0.7 %) with non-*P.falciparum* infections and four participants (4/888; 0.5 %) with mixed infections (*P. falciparum* and non-*P*. *falciparum;* Table 3). Using uPCR as reference, the sensitivity and specificity of RDTs were 28 and 100 %, respectively, in detecting *P. falciparum* infections and 3 and 99 % in detecting *P. vivax* infections (Table 4).

**Table 3:** *Plasmodium* species identification by RDT and qPCR

RDT	qPCR					
	Negative	*P. falciparum*	*P. vivax*	Mixed^a^	*Plasmodium* spp.	Total
Negative	708	23	96	13	30	870
*P. falciparum*	0	7	0	1	0	8
Non-falciparum	4	0	2	0	0	6
Mixed infection^a^	1	2	1	0	0	4
Total	713	32	99	14	30	888

^a^ *P. falciparum* and *P. vivax*

**Table 4:** RDT performance characteristics (sensitivity, specificity, positive predictive value, negative predictive value)

		RDT			
		Sensitivity % (95 % CI)	Specificity % (95 % CI)	PPV (95 % CI)	NPV (95 % CI)
uPCR	*P. falciparum* (n = 36)	27.8 (14.2–45.2)	99.9 (99.1–100)	90.9 (57.1–99.5)	96.5 (94.8–97.6)
	*P. vivax* (n = 109)	2.7 (0.7–8.2)	99.3 (98.3–99.7)	37.5 (10.2–74.1)	86.7 (84.1–88.9)

*PPV* positive predictive value, *NPV* negative predictive value

### Factors associated with *Plasmodium* infections

Five covariates were independently and significantly associated with *P. vivax* mono-infections (Table 5): male sex [Odds Ratio adjusted for age and district 4.76 (95 % CI 2.84–8.00)], increasing age [aOR 0.98 (95 % CI 0.96–0.99)], residing in Nong District [aOR 0.16 (95 % CI 0.10–0.27)], a history of three or more malaria episodes [aOR 1.56 (95 % CI 1.08–2.25)], and an overnight stay in a forest in the last 3 months [aOR 2.45 (95 % CI 1.17–5.11)]. There was a statistically significant association between a history of fever in the previous 48 h and *P. falciparum* mono-infections [aOR 2.47 (95 % CI 1.20–5.08; Table 6)]. Increasing age was found to be protective against *P. falciparum* infection in the univariate analysis [OR 0.97 (95 % CI 0.96–1.00)] but not in the multivariate analysis [aOR 0.98 (0.95–1.00)].

**Table 5:** Multiple logistic regression analysis to identify independently significant variables associated with *P. vivax* mono-infections (n = 99)

Covariates	Univariate analysis	P value	Multivariate analysis	P value
	Crude OR (95 % CI)		Adj. OR (95 % CI)	
Sex male (n = 812)	3.35 (2.06–5.46)	<0.001	4.76 (2.84–8.00)^a^	<0.001
Age (n = 812)	0.97 (0.96–0.99)	0.001	0.98 (0.96–0.99)^b^	0.002
District Nong (n = 812)	0.21 (0.13–0.34)	<0.001	0.16 (0.10–0.27)^c^	<0.001
History of Fever in the previous 48 h (n = 812)	0.78 (0.49–1.26)	0.31	1.02 (0.61–1.72)^d^	0.92
Feeling ill in the previous 48 h (n = 812)	0.71 (0.47–1.09)	0.12	0.98 (0.62–1.55)^d^	0.92
History of 3 or more malaria episodes (n = 802)	9.28 (4.96–17.36)	<0.001	1.56 (1.08–2.25)^d^	0.02
Stay overnight in the forest in the last 3 months (n = 812)	3.06 (2.00–4.70)	<0.001	2.45 (1.17–5.11)^d^	0.02
Travelled outside the village in the last 3 months (n = 812)	0.85 (0.51–1.39)	0.50	0.92 (0.54–1.57)^d^	0.76
Bed net never used (n = 812)	1.28 (0.58–2.79)	0.31	1.55 (0.66–3.66)^d^	0.45

^a^adjusted for age and district

^b^adjusted for sex and district

^c^adjusted for age and sex

^d^adjusted for age, sex and district

**Table 6:** Multiple logistic regression analysis to identify independently significant variables associated with *P. falciparum* mono-infections (n = 32)

Covariates	Univariate analysis	P value	Multivariate analysis	P value
	Crude OR (95 % CI)		OR adj. (95 % CI)	
Sex male (n = 745)	1.01 (0.50–2.06)	0.97	0.84 (0.87–4.65)^a^	0.65
Age (n = 745)	0.97 (0.96–1.00)	0.01	0.98 (0.95–1.00)^b^	0.08
District Nong (n = 745)	1.89 (0.84–4.3)	0.12	2.02 (0.87–4.65)^c^	0.10
History fever in the last 48 h (n = 745)	2.49 (1.22–5.08)	0.01	2.47 (1.20–5.08)^d^	0.01
Feeling ill in the last 48 h (n = 745)	1.55 (0.74–3.21)	0.24	1.70 (0.81–3.59)^d^	0.16
History of any malaria (n = 735)	1.75 (0.83–3.68)	0.14	1.68 (0.79–3.61)^d^	0.18
History of 3 or more malaria episodes (n = 735)	0.67 (0.09–5.30)	0.70	0.87 (0.10–7.40)^d^	0.90
Stay overnight in the forest in the previous 3 months (n = 745)	1.04 (0.47–2.28)	0.92	1.04 (0.44–2.41)^d^	0.94
Travelled outside the village in the previous 3 months (n = 745)	1.27 (0.59–2.73)	0.54	1.91 (0.55–2.58)^d^	0.66
Bed net never used (n = 745)	1.50 (0.44–5.11)	0.52	1.18 (0.34–4.10)^d^	0.80

^a^adjusted for age and district

^b^adjusted for sex and district

^c^adjusted for age and sex

^d^adjusted for age, sex and district

## Discussion

This is the first survey conducted in Laos of sub-clinical and sub-microscopic *Plasmodium* infections using uPCR. The study found that one-fifth of the afebrile population in southern Savannakhet was carrying *Plasmodium* infections, a third of which *P. falciparum* infections may be resistant to artemisinin derivatives. The prevalence of asymptomatic *Plasmodium* infection was very heterogeneously distributed not only between districts but also between villages separated by short distances.

There has been a shift from the traditional dominance of *P. falciparum,* which used to be the most prevalent (≥90 %) species in Laos, towards *P. vivax* [21–25]. An increase in the proportion of *P. vivax* infections has been reported from Thailand [26], Cambodia [27], Solomon Islands [28], Amazon [29, 30], and Central America [31] as falciparum malaria incidence has declined. A recent, large, multi-centre, cross-sectional survey conducted in Cambodia, Vietnam and Thai–Myanmar border found almost twice as many *P. vivax* infections compared to *P. falciparum* infections among a total of 988 *Plasmodium* infections [32]. Current malaria control practices in Laos are more effective in the control of *P. falciparum* than *P. vivax*. The complete, radical cure of vivax infections requires a 14-day course with 8-aminoquinolines, such as primaquine, and this is not implemented currently. There remains considerable reluctance to use primaquine in radical cure while G6PD testing is unavailable and there is therefore a significant haemolytic risk.

A high geographical heterogeneity was also found in the distribution of the K13 mutation C580Y which is associated with resistance against artemisinin derivatives [12, 19]. In Thapangthong District 75 % of the tested *P. falciparum* strains had the K13 C580Y mutation. In contrast, in Nong District the K13 mutation was only detected in 7 % of *P. falciparum* strains. This finding supports the hypothesis that with decreasing falciparum malaria transmission the more resistant strains replace wild type strains infections form an increasing proportion of the dwindling parasite population [14].

The heterogeneity in the prevalence of *Plasmodium* infections may at least in part be explained by environmental factors, such as the location of homes in relation to mosquito breeding sites, the design and construction materials of the home, and protective measures taken by the residents. The heterogeneity in the prevalence of *Plasmodium* infections in this study is consistent with an earlier study conducted in Laos [2]. The reason for heterogeneity suggested by the authors included the proximity of deep forest and the characteristics of the principal vectors in Laos which are *Anopheles dirus* and *Anopheles minimus* [33]. The dispersal range of *An. dirus* and *An. minimus,* combined with the anthropophilic behaviour of *An. minimus* was thought to explain at least some of the variability in malaria prevalence between villages [33, 34]. In a study conducted in Cambodia, villages proximal to forested areas had a higher prevalence of *Plasmodium* infections compared to villages more distant from forested areas [35]. Detailed observations on the micro-epidemiology of *P. falciparum* infections found similar large differences between villages in sub-Saharan Africa and in Europe in the last century [36]. Different environmental (mosquito densities, abundance of larval habitats) and human-mosquito behaviour (exposure to mosquitoes due to occupation, such as working in the forest) can explain in large part the geographical variation in species epidemiology [28]. Genetically determined host factors such as red cell abnormalities, and possibly immune response genes, may also contribute to differences observed between villages [37].

The sensitivity of RDTs in this study was 28 % for *P. falciparum* infections and 3 % for *P. vivax* infections using uPCR as reference. The wide discrepancy in prevalence measured by RDT compared to uPCR in this current study is consistent with a multi-national survey, which used the same uPCR detection technique [32]. This earlier study, conducted in Cambodia, Thai–Myanmar border and Vietnam, found a *Plasmodium* prevalence of 4 % by RDT compared to 20 % by uPCR. A survey using RDT conducted in Laos in 2006–2008 [1, 2] found a 1.2 % prevalence of *P. falciparum* infections—similar to the 0.9 % *P. falciparum* prevalence detected by RDT in the current study.

Several factors were associated with *P. vivax* mono-infections. Being a male was associated with higher prevalence of *P. vivax* infection than being female. The increased risk of contracting malaria for males may be explained by work-related exposure in the field and forested areas [3]. A similar gender imbalance was also observed on the Thailand-Myanmar border, in Cambodia [27] and Ethiopia [5, 38]. A history of sleeping in the forest was found to be associated with increased *P. vivax* prevalence. Forest workers have been traditionally a high-risk group for malaria in the Greater Mekong Sub-region [3, 39–43]. Control of forest malaria has proven to be highly challenging and innovative approaches will be needed to interrupt malaria transmission among forest workers.

The peak risk for *Plasmodium* infections in this study was in the age group 15–35 years and decreased with older age. Younger participants may have been less exposed to vectors while older participants may have developed sufficient immunity to clear infections. A similar peak prevalence in adults has been observed in past studies in Laos [44] and Cambodia [35].

## Conclusion

The study found a high prevalence of asymptomatic *Plasmodium* infections as detected by uPCR and three times more *P. vivax* than *P. falciparum* infections in the southern Savannakhet Province. The distribution of infections was very heterogeneous, with some villages nearly free of infections while in other villages more than half of the village population was infected. The majority of *P. falciparum* strains in Thapangthong District carried markers suggesting reduced susceptibility to artemisinin derivatives. These strains are also already present in the more remote Nong District. This worrying trend has wider implications for Laos and could reverse the gains achieved by the successful control of malaria in Laos and the GMS. The rapid elimination of *P. falciparum* should be a top priority in Laos and the GMS before malaria there becomes untreatable. Such elimination will require targeted interventions in high prevalence villages.
